# Supplementary material for: Investigating In Vitro and Ex Vivo Properties of Artemether/Lumefantrine Double-Fixed Dose Combination Lipid Matrix Tablets Prepared by Hot Fusion
Source: Pharmaceutics. 2021 Jun 22;13(7):922. doi: 10.3390/pharmaceutics13070922 (PMC8308765; doi:10.3390/pharmaceutics13070922)
Supplement: Supplementary file 1 [file pharmaceutics-13-00922-s001.zip › pharmaceutics-1213028-SI.pdf]

pharmaceutics MDPI

Supplementary Materials: Investigating In Vitro and Ex Vivo Properties of Artemether/Lumefantrine Double-Fixed Dose Combination Lipid Matrix Tablets Prepared by Hot Fusion

Christi A. Wilkins, Lissinda H. du Plessis and Joe M. Viljoen

S1. Physical Properties of the Different Lipid Matrix Tablet Formulations Analyzed

During preformulation studies, it was concluded that neither RetaLac® (R) nor Pharmacel® 101 (P) were suitable filler options for the manufacture of lipid matrix tablets as neither produced lipid matrix tablets with the necessary physical tablet characteristics to meet the test criteria utilizing the given factors of this study. It is important to note that whilst glycerol monostearate and stearic acid formulations containing either RetaLac® or Pharmacel® 101 produced poor tablets, cetyl alcohol in combination with these fillers produced no tablets, indicating that neither are viable lipid base-filler combinations.

All Pharmacel® 101 containing formulations, irrespective of the lipid base, experienced incomplete filling of the tablet die during tableting. This in turn resulted in relatively high percentage relative standard deviations (%RSDs) during mass variation trials (Table A2). From Table A3, it is clear that Pharmacel® 101 depicted the highest %RSD for mass variation (3.758%) followed by RetaLac® (2.708%). Formulation SA0.5P1.25 portrayed the highest percentage friability of 77.85% (Table A2) and only 3 of the 18 Pharmacel® 101 formulations, namely GM0.75P1, GM1P1.25 and SA0.75P1, met the friability criteria. Pharmacel® 101 produced tablets with markedly low tensile strength, on average 0.309 $N.mm^{-2}$, and several tablet formulations (disregarding the lipid base or lipid:drug ratio) crumbled upon handling. Many of these lipid matrix tablets were too soft to obtain any hardness or tensile strength results and subsequently no clear conclusions could be drawn.

RetaLac® formulations depicted the second highest %RDS during mass variation assessment (2.708%) as well as the lowest average tensile strength (0.172 $N.mm^{-2}$). RetaLac® lipid matrix tablets portrayed an average friability of 12.460%, with GM0.5R1.25 demonstrating the highest friability (76.022%). Unfortunately, despite RetaLac® formulations passing the disintegration tests and forming a gel matrix system, the lack of appropriate tablet properties such as hardness and friability, resulted in its inability to produce lipid matrix tablets of suitable integrity, rendering it inappropriate for further investigation.

It was furthermore observed that when all the factors were considered and the averages calculated per factor (Table A3), the poor results of RetaLac®and Pharmacel® 101 lipid matrix tablet formulations were skewing the overall data obtained. Therefore, a fair and accurate comparison could not be conducted. For this reason, these fillers were excluded from the main reporting.

The inclusion of magnesium stearate at the two levels of 1% and 1.25% did not produce a substantial difference in results between formulations containing MicroceLac® 100 and CombiLac® as seen in Table 2 in article text. The higher level (1.25%) was however necessary during the production of formulations containing RetaLac® and Pharmacel® 101, as it reduced tablet adherence to the punches during tableting and yielded more aesthetically appealing tablets overall. The influence of lubricant concentration on these formulations is clearly demonstrated in Figure B4.

Table S1. Average values obtained for the physical properties (mass variation presented as %RSD in parenthesis) of the lipid matrix tablet formulations containing either MicroceLac® or CombiLac® as filler. Values highlighted and in bold indicate that the formulation did not adhere to the criteria for the specific experiment.

		Test	Unit	Stearic acid (SA)			Glycerol monostearate (GM)			Cetyl alcohol (CA)		
				0.5:1	0.75:1	1:1	0.5:1	0.75:1	1:1	0.5:1	0.75:1	1:1
MicroceLac®	1% MgSt	Mass variation	%	(0.91)	(1.15)	(0.85)	(1.47)	(1.94)	(0.92)	(1.55)	(1.85)	(1.89)
		Friability	%	0.153	0.121	0.122	0.143	0.180	0.136	0.997	0.998	0.999
		Crushing strength	N	218.9	132.3	140.6	162.6	143.7	155.0	157.8	182.5	185.8
		Tensile strength	N.mm^{-2}	2.393	1.351	1.464	1.856	1.515	1.738	1.863	2.169	2.196
		Disintegration	min	ND	ND	ND	ND	ND	ND	ND	ND	ND
	1.25% MgSt	Mass variation	%	(0.94)	(0.87)	(0.72)	(1.14)	(1.73)	(1.31)	(1.96)	(2.04)	(1.49)
		Friability	%	0.181	0.121	0.101	0.100	0.162	0.226	0.994	0.998	0.995
		Crushing strength	N	244.5	159.6	129.3	156.1	140.4	160.6	162.2	179.2	166.2
		Tensile strength	N.mm^{-2}	2.859	1.693	1.372	1.785	1.540	1.903	1.992	2.071	1.903
		Disintegration	min	ND	ND	ND	ND	ND	ND	ND	ND	ND
CombiLac®	1% MgSt	Mass variation	%	(2.30)	(0.98)	(0.71)	(1.74)	(1.47)	(1.06)	(1.71)	(1.61)	(1.29)
		Friability	%	0.234	0.060	0.800	0.114	0.040	0.181	0.998	0.999	0.999
		Crushing strength	N	257.8	145.3	172.2	153.4	170.0	150.1	176.9	162.0	156.9
		Tensile strength	N.mm^{-2}	2.886	1.532	1.817	1.563	1.854	1.521	2.073	3.259	1.758
		Disintegration	min	ND	ND	ND	ND	10.68	14.13	ND	ND	ND
	1.25% MgSt	Mass variation	%	(1.02)	(0.92)	(0.74)	(8.28)	(0.71)	(1.04)	(1.83)	(1.66)	(1.75)
		Friability	%	0.283	0.183	0.243	0.313	0.297	0.612	0.998	0.998	0.996
		Crushing strength	N	244.6	224.7	151.8	86.8	164.6	151.6	165.4	177.9	158.0
		Tensile strength	N.mm^{-2}	2.874	2.535	1.601	0.928	1.810	1.705	1.931	2.114	1.839
		Disintegration	min	ND	13.40	ND	ND	ND	14.44	ND	ND	ND

Table S2. Average values obtained for the physical properties (mass variation presented as %RSD in parenthesis) of the lipid matrix tablet formulations containing either Pharmacel®101 or RetaLac® as filler. Values highlighted and in bold indicate that the formulation did not adhere to the criteria for the specific experiment.

		Test	Unit	Stearic acid (SA)			Glycerol monostearate (GM)			Cetyl alcohol (CA)		
				0.5:1	0.75:1	1:1	0.5:1	0.75:1	1:1	0.5:1	0.75:1	1:1
Pharmacel®101	1% MgSt	Mass variation	%	(3.77)	(1.22)	(2.88)	(1.71)	(3.67)	(4.18)			
		Friability	%	57.73	3.682	10.722	1.218	0.552	0.521			
		Crushing strength	N	0	20.600	15.400	38.700	49.700	67.100	NR	NR	NR
		Tensile strength	N.mm^{-2}	0	0.152	0.116	0.347	0.457	0.628			
		Disintegration	min	0.59	0.56	2.04	0.36	0.26	0.33			
	1.25% MgSt	Mass variation	%	(7.66)	(2.62)	(2.23)	(6.03)	(7.28)	(1.85)			
		Friability	%	77.85	0.429	1.869	10.127	2.319	0.269			
		Crushing strength	N	0	43.200	26.000	27.111	69.625	82.100	NR	NR	NR
		Tensile strength	N.mm^{-2}	0	0.374	0.193	0.206	0.514	0.720			
		Disintegration	min	0.55	0.64	1.08	0.34	0.61	0.43			
RetaLac®	1% MgSt	Mass variation	%	(1.07)	(1.91)	(1.67)	(4.11)	(2.37)	(6.44)			
		Friability	%	3.149	3.149	1.127	12.487	10.322	1.005			
		Crushing strength	N	36.800	19.100	26.800	0	13.286	52.250	NR	NR	NR
		Tensile strength	N.mm^{-2}	0.333	0.147	0.202	0	0.079	0.400			
		Disintegration	min	ND	ND	ND	ND	ND	ND			
	1.25% MgSt	Mass variation	%	(1.50)	(1.30)	(3.15)	(4.44)	(1.65)	(2.88)			
		Friability	%	10.822	10.822	1.840	76.022	14.653	4.118			
		Crushing strength	N	27.700	21.900	25.800	0	12.667	24.889	NR	NR	NR
		Tensile strength	N.mm^{-2}	0.232	0.173	0.203	0	0.093	0.204			
		Disintegration	min	ND	ND	ND	ND	ND	ND			

MgSt = Magnesium stearate; ND = Non-disintegrating; NR = No Results.

Table S3. Comparison of all formulation factors, including RetaLac® and Pharmacel®101 fillers, investigated in the full factorial design regarding average mass variation, friability and tensile strength results to demonstrate the negative effect of these fillers on reporting.

Factors	Mass variation		Friability	Crushing Strength		Tensile Strength	
	Mass (mg)	%RSD	(%)	(N)	%RSD	(N.mm^{-2})	%RSD
MicroceLac®100	494.939	1.390	0.429	165.406	19.611	1.850	0.148
RetaLac®	487.692	2.708	12.460	21.766	25.470	0.172	0.278
CombiLac®	494.533	1.710	0.519	170.556	20.400	1.978	0.216
Pharmacel® 101	497.804	3.758	13.941	36.628	24.828	0.309	0.249
Stearic Acid	499.783	1.027	0.217	93.014	24.490	2.031	0.249
Glycerol Monostearate	498.592	1.912	0.209	103.538	25.886	1.613	0.086
Cetyl Alcohol	485.833	1.719	0.997	169.233	12.528	2.097	0.211
0.5:1	495.775	2.088	0.459	109.922	11.078	2.084	0.142
0.75:1	496.500	1.411	0.430	115.866	27.746	1.923	0.169
1:1	491.930	1.158	0.534	111.614	21.192	1.735	0.235
MgSt 1%	497.130	1.411	0.460	112.118	20.497	1.914	0.216
MgSt 1.25%	492.339	1.694	0.489	112.816	19.514	1.914	0.148

MgSt = Magnesium stearate; ND = Non-disintegrating.

S2. Photographs of internal and external tablet morphologies for the various lipid matrix tablet formulations

Table S4. Abbreviations allocated to formulations.

Abbreviation	Lipid	Lipid:drug ratio	Filler	Lubricant concentration
SA0.5M1	Stearic acid;	0.5:1 lipid:drug ratio;	MicroceLac®100;	1% Magnesium stearate
SA0.5C1	Stearic acid;	0.5:1 lipid:drug ratio;	CombiLac®;	1% Magnesium stearate
SA0.75M1	Stearic acid;	0.75:1 lipid:drug ratio;	MicroceLac®100;	1% Magnesium stearate
SA0.75C1	Stearic acid;	0.75:1 lipid:drug ratio;	CombiLac®;	1% Magnesium stearate
SA1M1	Stearic acid;	1:1 lipid:drug ratio;	MicroceLac®100;	1% Magnesium stearate
SA1C1	Stearic acid;	1:1 lipid:drug ratio;	CombiLac®;	1% Magnesium stearate
GM0.5M1	Glycerol monostearate;	0.5:1 lipid:drug ratio;	MicroceLac®100;	1% Magnesium stearate
GM0.5C1	Glycerol monostearate;	0.5:1 lipid:drug ratio;	CombiLac®;	1% Magnesium stearate
GM0.75M1	Glycerol monostearate;	0.75:1 lipid:drug ratio;	MicroceLac®100;	1% Magnesium stearate
GM0.75C1	Glycerol monostearate;	0.75:1 lipid:drug ratio;	CombiLac®;	1% Magnesium stearate
GM1M1	Glycerol monostearate;	1:1 lipid:drug ratio;	MicroceLac®100;	1% Magnesium stearate
GM1C1	Glycerol monostearate;	1:1 lipid:drug ratio;	CombiLac®;	1% Magnesium stearate
CA0.5M1	Cetyl alcohol;	0.5:1 lipid:drug ratio;	MicroceLac®100;	1% Magnesium stearate
CA0.5C1	Cetyl alcohol;	0.5:1 lipid:drug ratio;	CombiLac®;	1% Magnesium stearate
CA0.75M1	Cetyl alcohol;	0.75:1 lipid:drug ratio;	MicroceLac®100;	1% Magnesium stearate
CA0.75C1	Cetyl alcohol;	0.75:1 lipid:drug ratio;	CombiLac®;	1% Magnesium stearate
CA1M1	Cetyl alcohol;	1:1 lipid:drug ratio;	MicroceLac®100;	1% Magnesium stearate
CA1C1	Cetyl alcohol;	1:1 lipid:drug ratio;	CombiLac®;	1% Magnesium stearate
GM0.5P1	Glycerol monostearate;	0.5:1 lipid:drug ratio;	Pharmacel®101;	1% Magnesium stearate
GM0.5P1.25	Glycerol monostearate;	0.5:1 lipid:drug ratio;	Pharmacel®101;	1.25% Magnesium stearate
GM0.75P1	Glycerol monostearate;	0.75:1 lipid:drug ratio;	Pharmacel®101;	1% Magnesium stearate
GM0.75P1.25	Glycerol monostearate;	0.75:1 lipid:drug ratio;	Pharmacel®101;	1.25% Magnesium stearate
GM1P1	Glycerol monostearate;	1:1 lipid:drug ratio;	Pharmacel®101;	1% Magnesium stearate
GM1P1.25	Glycerol monostearate;	1:1 lipid:drug ratio;	Pharmacel®101;	1.25% Magnesium stearate
GM0.75R1	Glycerol monostearate;	0.75:1 lipid:drug ratio;	RetaLac®;	1% Magnesium stearate
GM0.75R1.25	Glycerol monostearate;	0.75:1 lipid:drug ratio;	RetaLac®;	1.25% Magnesium stearate

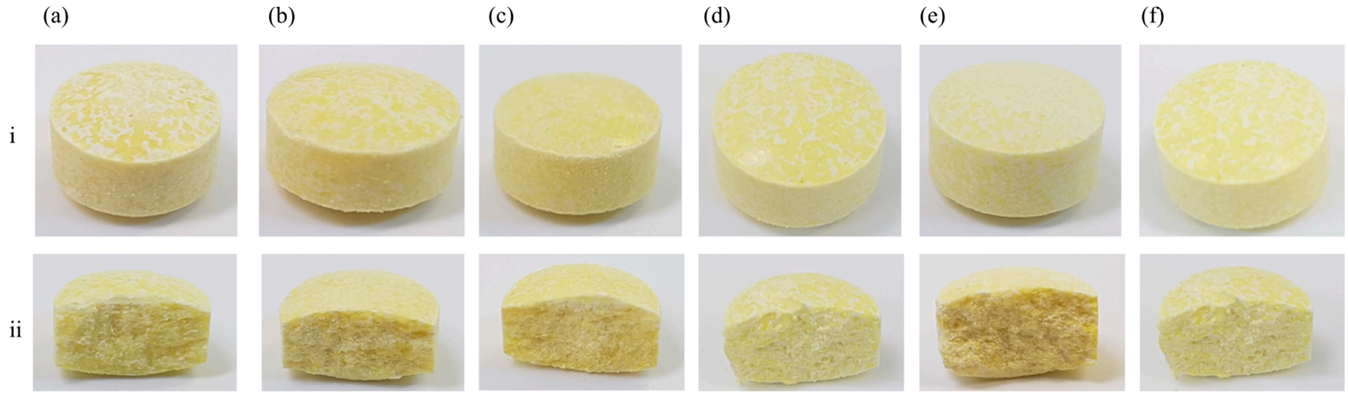

Figure S1. Morphology photographs showing (**i**) external and (**ii**) internal surfaces for formulations (**a**) SA0.5M1; (**b**) SA0.5C1; (**c**) SA0.75M1; (**d**) SA0.75C1; (**e**) SA1M1; and (**f**) SA1C1.

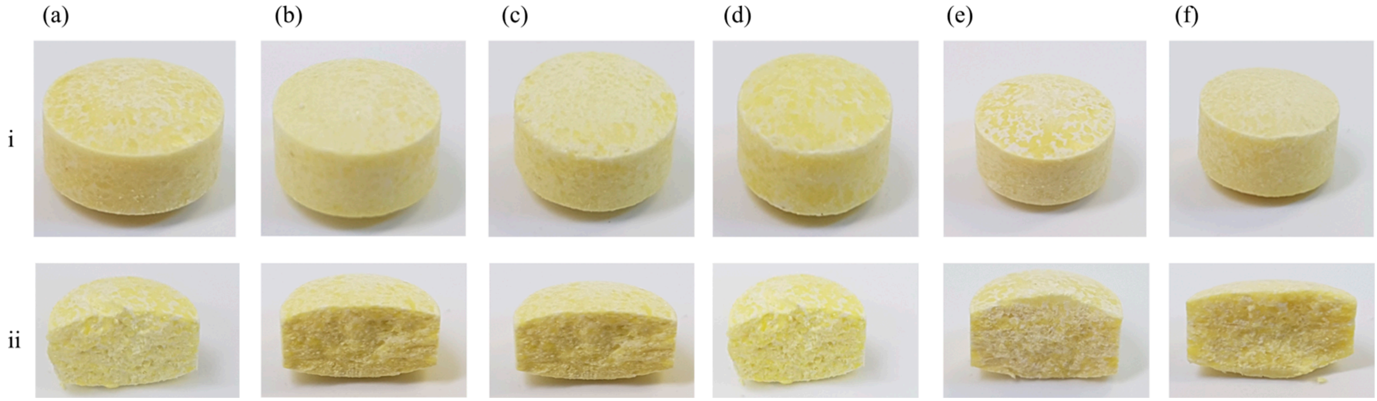

Figure S2. Morphology photographs showing (**i**) external and (**ii**) internal surfaces for formulations (**a**) GM0.5M1; (**b**) GM0.5C1; (**c**) GM0.75M1; (**d**) GM0.75C1; (**e**) GM1M1; and (**f**) GM1C1.

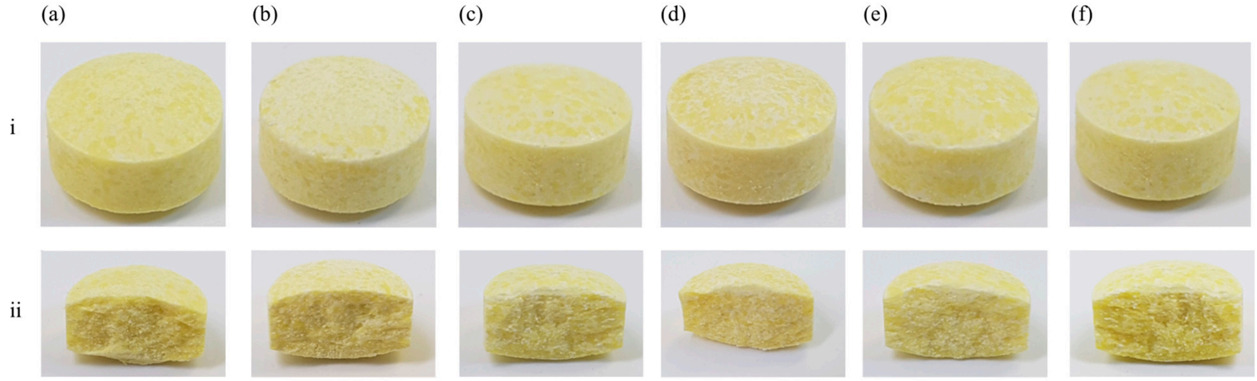

Figure S3. Morphology photographs showing (**i**) external and (**ii**) internal surfaces for formulations (**a**) CA0.5M1; (**b**) CA0.5C1; (**c**) CA0.75M1; (**d**) CA0.75C1; (**e**) CA1M1; and (**f**) CA1C1.

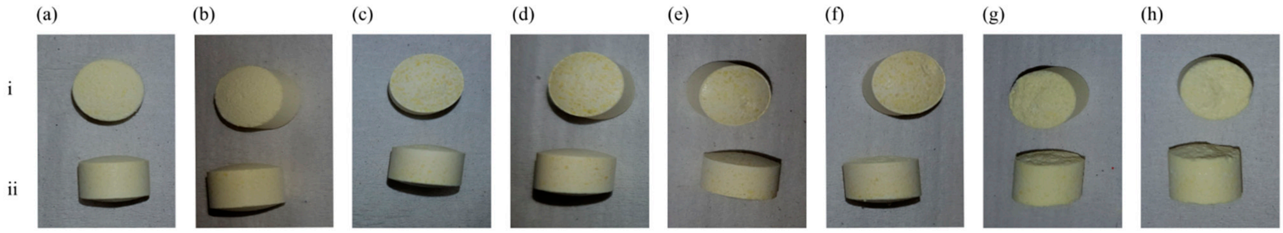

Figure S4. Morphology photographs showing (i) top view and (ii) side view of tablets for selected formulations (**a**) GM0.5P1; (**b**) GM0.5P1.25; (**c**) GM0.75P1; (**d**) GM0.75P1.25; (**e**) GM1P1; (**f**) GM1P1.25 (**g**) GM0.75R1 and (**h**) GM0.75R1.25; where P signifies Pharmacel®101 and R signifies RetaLac®.

S3. Assay: Artemether and Lumefantrine Content Determined

ART and LUM content were tested per formulation. As described, the lipid matrix tablets were formulated to contain 120 mg LUM and 20 mg ART per tablet. The results pertaining to the assay experiments are recorded in Table C.1 and presented as the calculated %API content per formulation. Additionally, when considering lumefantrine formulations, it was seen to demonstrate a %RSD-value of 20.3% across all variables; whilst artemether displayed a %RSD-value of 27.1% across all investigated variables.

Table S5. %API content determined per formulation.

%Artemether	Formulation	%Lumefantrine
95.00	SA1C1	93.20
78.54	SA1M1	91.60
91.50	SA0.5C1	105.46
100.56	SA0.5M1	116.28
72.43	SA0.75C1	131.72
75.34	SA0.75M1	133.37
60.21	GM1C1	72.79
104.20	GM1M1	101.60
67.23	GM0.5C1	74.74
100.77	GM0.5M1	116.56
86.64	GM0.75C1	98.15
82.66	GM0.75M1	91.30
71.24	CA1C1	86.16
65.67	CA1M1	80.93
99.34	CA0.5C1	112.54
126.10	CA0.5M1	117.60
81.31	CA0.75C1	92.94
79.92	CA0.75M1	89.84

S4. Fit Factors: Indicating Similarities and Differences Between Tested Formulations

Table S6. Fit factors analyzed indicating statistically significant differences in green and similarities in red.

Formulation		CA0.5M1	CA0.75M1	CA1M1	CA0.5C1	CA0.75C1	CA1C1	SA0.5M1	SA0.75M1	SA1M1	SA0.5C1	SA0.75C1	SA1C1	GM0.5M1	GM0.75M1	GM1M1	GM0.5C1	GM0.75C1	GM1C1
CA0.5M1	f1		36.9	37.7	36.0														
	f2		38.3	40.3	43.2														
CA0.75M1	f1	36.9		83.4		44.0													
	f2	38.3		30.0		42.3													
CA1M1	f1	37.7	83.4				48.4												
	f2	40.3	30.0				31.1												
CA0.5C1	f1	36.0				4.6	37.3												
	f2	43.2				82.2	40.6												
CA0.75C1	f1		44.0		4.6		34.2												
	f2		42.3		82.2		43.3												
CA1C1	f1			48.4	37.3	34.2													
	f2			31.1	40.6	43.3													
SA0.5M1	f1								16.7	19.4	3.0								
	f2								55.3	50.4	85.6								
SA0.75M1	f1							16.7		16.1		18.7							
	f2							55.3		58.7		56.1							
SA1M1	f1							19.4	16.1				8.5						
	f2							50.4	58.7				71.4						
SA0.5C1	f1							3.0				5.4	21.3						
	f2							85.6				77.1	48.5						
SA0.75C1	f1								18.7		5.4		21.6						
	f2								56.1		77.1		47.4						
SA1C1	f1									8.5	21.3	21.6							
	f2									71.4	48.5	47.4							
GM0.5M1	f1														42.7	17.2	13.4		
	f2														36.4	57.7	62.5		
GM0.75M1	f1													42.7		89.1		81.6	
	f2													36.4		32.1		34.0	

GM1M1	f1	17.2	89.1				65.1
	f2	57.7	32.1				23.7
GM0.5C1	f1	13.4				12.4	67.0
	f2	62.5				58.8	24.1
GM0.75C1	f1		81.6		12.4		69.9
	f2		34.0		58.8		25.7
GM1C1	f1			65.1	67.0	69.9	
	f2			23.7	24.1	25.7	

1
